# Spatio-Temporal Analysis of Influenza-Like Illness and Prediction of Incidence in High-Risk Regions in the United States from 2011 to 2020

**DOI:** 10.3390/ijerph18137120

**Published:** 2021-07-02

**Authors:** Zhijuan Song, Xiaocan Jia, Junzhe Bao, Yongli Yang, Huili Zhu, Xuezhong Shi

**Affiliations:** 1 Department of Epidemiology and Biostatistics, College of Public Health, Zhengzhou University, Zhengzhou 450001, China; zjsong178@163.com (Z.S.); jxc881223@126.com (X.J.); baojz@zzu.edu.cn (J.B.); ylyang377@zzu.edu.cn (Y.Y.); zhuhuili39@163.com (H.Z.); 2 Zhengzhou University Library, Zhengzhou University, Zhengzhou 450001, China

**Keywords:** influenza-like illness, spatiotemporal analysis, SARIMA model, prediction

## Abstract

About 8% of the Americans contract influenza during an average season according to the Centers for Disease Control and Prevention in the United States. It is necessary to strengthen the early warning for influenza and the prediction of public health. In this study, Spatial autocorrelation analysis and spatial scanning analysis were used to identify the spatiotemporal patterns of influenza-like illness (ILI) prevalence in the United States, during the 2011–2020 transmission seasons. A seasonal autoregressive integrated moving average (SARIMA) model was constructed to predict the influenza incidence of high-risk states. We found the highest incidence of ILI was mainly concentrated in the states of Louisiana, District of Columbia and Virginia. Mississippi was a high-risk state with a higher influenza incidence, and exhibited a high-high cluster with neighboring states. A SARIMA (1, 0, 0) (1, 1, 0)_52_ model was suitable for forecasting the ILI incidence of Mississippi. The relative errors between actual values and predicted values indicated that the predicted values matched the actual values well. Influenza is still an important health problem in the United States. The spread of ILI varies by season and geographical region. The peak season of influenza was the winter and spring, and the states with higher influenza rates are concentrated in the southeast. Increased surveillance in high-risk states could help control the spread of the influenza.

## 1. Introduction

Influenza is caused by the influenza virus which mainly spreads through airborne droplets and direct contact. It has the characteristics of strong infectivity, rapid transmission and antigen variation. The activity of seasonal influenza begins to increase in October, most often peaks between December and February and can remain elevated until May. Influenza virus infections are very common and their incidence can only be estimated [1]. Previous estimates attributed to the World Health Organization indicated that 250,000–500,000 influenza-associated deaths occur annually, corresponding to estimates of 3.8–7.7 deaths per 100,000 individuals calculated using 2005 United Nations Department of Economic and Social Affairs World Population Prospects [2]. In particular, the 2017–2018 influenza season in the United States was notable for its high severity, with about 45 million illnesses and 810,000 influenza-associated hospitalizations throughout the United States [2].

As influenza may be characterized by fever, cough, sore throat, runny or stuffy nose, body aches, headache, chills or fatigue and so on, it is hard to diagnose as influenza, based on symptoms alone. A number of influenza tests are available to detect influenza viruses in respiratory specimens. The most common are called “rapid influenza diagnostic tests (RIDTs)” [3]. However, not all the people were tested for influenza, the number of reported cases of influenza may significantly underestimate the actual prevalence of influenza. A prospective study in two metropolitan areas of Taiwan showed that influenza-like symptoms were significant predictors for influenza infection. The combination of fever plus cough had the best sensitivity (86%), but the combination of fever plus cough and sneeze had the best specificity (77%) [4]. Therefore, influenza-like illness (ILI) can be used instead of influenza to estimate the trend of influenza [5].

Influenza activity varied widely across the country, making the use of national burden estimates difficult for state or county public health messaging, planning, and responses [2]. A study of the temporal and spatial distribution of weekly influenza in Pennsylvania suggested that there was spatial heterogeneity and spatiotemporal aggregation of influenza distribution [6]. Martin [7] used spatiotemporal statistical prediction models to estimate daily ILI-related emergency department visits. The Geographic Information System (GIS) is a useful epidemiological method for identifying high-risk areas in many infectious diseases [8]. Global and local Moran’s I spatial autocorrelation analyses are common methods to detect whether there is spatial autocorrelation and where the specific areas are located, separately. Spatiotemporal scanning statistics is a more comprehensive method that can analyze space and time distribution and set parameters more flexibly [9]. Identifying high-risk and endemic areas and the spatial−temporal distribution of infectious diseases is important for the development of prevention plans and health policies [10].

The prevalence of influenza displays a seasonal pattern [11]. For diseases that show recurrent seasonal patterns or occur in cyclic patterns, time-series models are the most widely used statistical models for forecasting [12]. The autoregressive integrated moving average (ARIMA) is a widely used predictive analysis model for nonstationary time-series [13]. Compared with ARIMA model, the seasonal autoregressive integrated moving average (SARIMA) model considers seasonal characteristics and can more accurately identify the fluctuation of the diseases over time. The SARIMA model has been used to predict influenza in many studies. Yuzhou [14] used the SARIMA model to control the effects of seasonality in the forecast of influenza epidemics, and the results showed that the SARIMA model had better predictive performance. Xin [15] constructed several SARIMA models to predict the incidence of influenza in different provinces in China, and concluded that time-series analysis was good tool for the prediction of disease incidence.

This study used spatial autocorrelation analysis and spatiotemporal scanning analysis to identify the spatiotemporal patterns of ILI epidemics in the United States, during the 2011–2020 transmission seasons. A time-series model was then constructed, based on the influenza data of high-risk states, to predict the future incidence of influenza, so as to provide theoretical guidance and scientific basis for the prevention and treatment of influenza [16].

## 2. Materials and Methods

### 2.1. Data Resources

Information on outpatient visits to health care providers for ILI is collected weekly through the United States Outpatient Influenza-like Illness Surveillance Network. For this system, the confirmed influenza case was “A patient who tests positive for influenza virus infection by an approved laboratory test”, and ILI is defined as “fever (temperature of 100 °F (37.8 °C) or greater) and a cough and/or a sore throat without a known cause other than influenza” (https://www.cdc.gov/flu/weekly/overview.htm, accessed on 24 November 2020).

We collected the ILI data from 1st week 2011 to 29th week 2020 from the Centers for Disease Control and Prevention (CDC). The data included the number of ILI cases in 49 states for different age groups. For hotspot states, time-series models were constructed by collecting data from 1st week 2011 to 52nd week 2018, and data from 1st week 2019 to 29th week 2020 were taken as test data to assess forecast performance [17].

### 2.2. Spatiotemporal Cluster Analysis

Moran’s I is an important index for analyzing the spatial correlation of diseases [18]. Moran’s I ranges from −1 to 1, where 0 indicates a random distribution of influenza. A value close to 1 indicates that the unit cluster has a similar value. A value close to −1 indicates that the unit with high values and low values are adjacent in space [19]. Based on its value and significance, Moran’s I can detect four types of cluster, including the high-high (HH), high-low (HL), low-low (LL) and low-high (LH) clustering patterns, respectively. The number of permutations was 999, and the significance level was 0.05 [20].

Spatiotemporal cluster analysis is a measurement of temporal and spatial correlation on the foundation of spatial autocorrelation with the further consideration of the time factor [21]. It can relate the spatial characteristics to the temporal characteristics of influenza [16]. During the study period, the cluster was detected by retrospective spatiotemporal permutation scanning statistics [22]. A retrospective study is an analysis of a fixed geographic area and research period. The satellite scanning software scans multiple start and end dates, and evaluates real-time clusters (continuing to the study period and date) and historical clusters (which did not exist before the end date of the study period). Spatiotemporal scan statistics are defined by a specific window with a circular geographic base and height corresponding to time. The window size was constantly adjusted to detect possible spatiotemporal clusters [22]. In order to scan for small to large clusters, the largest radius was set to 50% of the total population at risk, the largest height was set to 50% of the total study period [23]. The logarithmic likelihood ratio (LLR) was used to compare observed and expected numbers to identify specific clusters. After detecting the most likely spatiotemporal clusters, these clusters were tested by the Monte Carlo method [24].

Monte Carlo simulation generates random copies of the data set under appropriate null hypotheses to determine the statistical significance of these results. The *p* values for these tests are calculated by comparing the maximum likelihood levels from the real data set with the maximum likelihood levels from the random data set, where *p* = rank/(1 + number of simulations) [11]. The number of copies should be at least 999 times to ensure sufficient accuracy. Therefore, we use 999 Monte Carlo replications to estimate the importance level of these clusters. If the points conforming to the evaluated cluster maintained their aggregated pattern when compared with 999 randomized simulations of the entire dataset, then it was considered important [25].

### 2.3. Time-Series Analysis

Time-series analysis has the advantage of predicting incidence. It is characterized by the number of patients in the past and responds by predicting the number of patients in the future. The SARIMA model is based on the sequential lag relationship existing in time-series data and more suitable for forecasting when the data has obvious seasonal characteristics [13]. The SARIMA model can be expressed as: SARIMA (p, d, q) (P, D, Q)_s_. Letters p, d, q are the order of autoregression, the order of difference and the order of moving average; Letters P, D, Q are the order of seasonal autoregression, the order of difference and the order of moving average, and s is the specific value of cycle, the cycle of American influenza is 52 weeks (s = 52) [13].

The process of establishing the SARIMA model was divided into three steps: First, a weekly time-series plot of incidence (per 100,000 population) was drawn to check for stationarity and seasonality. The model was constructed according to the autocorrelation function (ACF) and partial autocorrelation function (PACF) of the model residuals. Secondly, ACF and PACF for estimating residuals were tested by Ljung-Box Q test, and the minimum of the Bayesian information criterion (BIC) was taken as the optimal SARIMA model. Finally, the model was applied to forecast the weekly ILI incidence for 30th week 2020 to 52nd week 2021.

### 2.4. Statistical Analysis

The data was organized by Microsoft Excel 2013. The SARIMA model was constructed by R 3.6.0 and SPSS 27.0. The value of Moran’s I and local indicators of spatial association were calculated by GeoDa 1.14.0. The time scan statistic was measured with SaTScan^TM^9.5. All the maps were drawn by ArcGIS 10.0.

## 3. Results

### 3.1. Epidemiological Analysis

Included in our study were a total of 9,065,910 ILI cases from 1st week 2011 to 29th week 2020 in the United States. The ILI annual infection rate fluctuated from 5.92 to 15.84 per 100,000 population. ILI occurred throughout the year, most often peaked between December and February and lasted until May.

**Figure 1:**
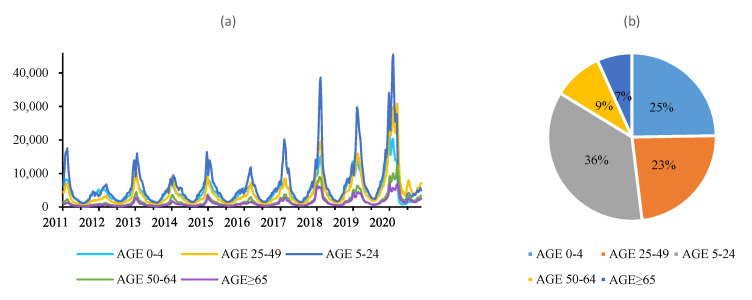
Epidemiological characteristics of influenza and ILI in the United States from 1st week 2011 to 29th week 2020. (**a**) Weekly ILI cases of different age, (**b**) age distribution of ILI.

In terms of age, the number of ILI cases in the age group 5–24 years old was the most, and these groups accounted for about 35 percent, while the number of patients in the age group over 65 years old was the least, accounting for about 7 percent (Figure 1). The difference between different age groups had a statistical significance (*p* < 0.001).

**Figure 2:**
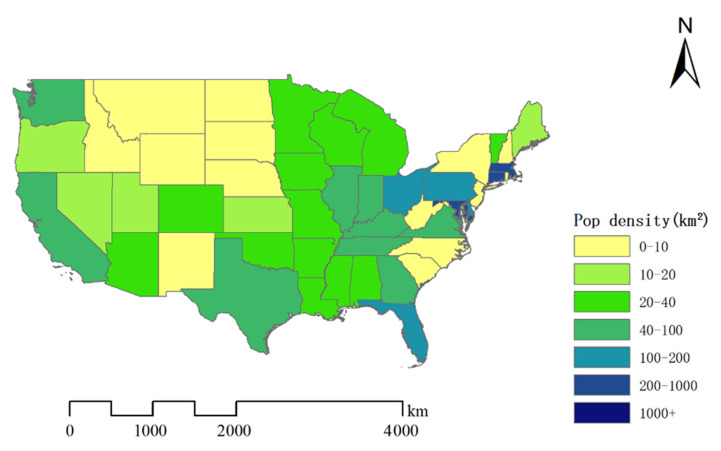
Map of population density by state in the United States. The map was created by ArcGIS software.

This study collected the population of 49 states and visualized them on the map (Figure 2), and found no obvious association between population density and influenza incidence.

### 3.2. Spatiotemporal Analysis

**Figure 3:**
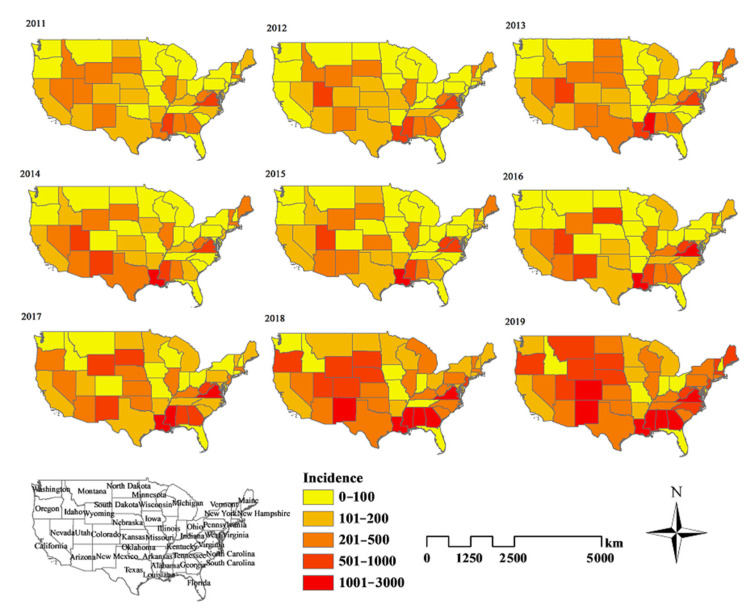
Maps of ILI incidence for each state in the United States from 2011 to 2019 by ArcGIS software.

Overall, the highest cumulative incidence of ILI (per 100,000 population) during the study period was seen in the states of Louisiana, District of Columbia and Virginia, which reported 12,200, 9563 and 9554 cases, respectively. The lowest cumulative incidence of ILI incidence was reported from the states of Ohio, Washington and Iowa (Figure 3).

#### 3.2.1. Global Spatial Autocorrelation

The global spatial autocorrelation analysis for ILI suggested a clustering distribution at the state level in the years of 2012 to 2017, the global Moran’s I reached the significance level of 0.05. In contrast, the global Moran’s I for 2011, 2018 and 2019 display no significant spatial autocorrelation, though Moran’s I greater than 0 (Table 1).

#### 3.2.2. Local Spatial Autocorrelation

**Figure 4:**
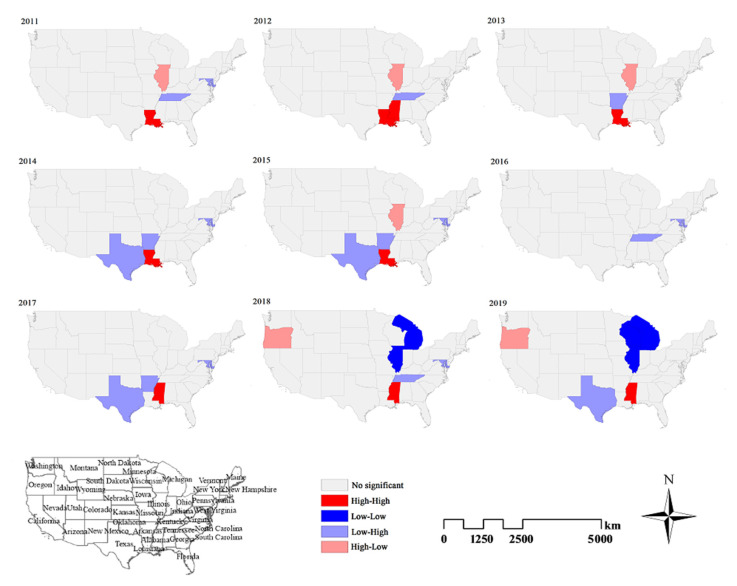
Maps of local spatial autocorrelation cluster of ILI for each state in the United States from 2011 to 2019 by GeoDa software.

Local spatial autocorrelation analysis reveals only the relative states, rather than absolute correlations. Only those states whose local Moran’s I have reached the significance level of 0.05 will be present on the map. From 2011 to 2019, the local spatial autocorrelation showed three HH clusters in total with two HL clusters, four LH and three LL clusters. HH clusters were observed in the states of Louisiana (5 years), Mississippi (4 years), and District of Columbia (1 year). Louisiana and Mississippi had HH clusters for long periods. HL clusters were observed in the states of Illinois (4 years), and Oregon (2 years). LH clusters were observed in the states of Tennessee (4 years), Maryland (6 years), Arkansas (5 years), and Texas (3 years). LL clusters appeared in the northeastern part of the United States only in 2018 and 2019 (Figure 4).

#### 3.2.3. Spatiotemporal Cluster Analysis

The spatiotemporal cluster analysis detected 23 clusters of ILI in the study period. The clusters were particularly obvious in spring and winter. For example, the risk ratio (RR) was highest in 2015, with three levels of clustering. Level 1, with Louisiana at the center of high incidence area and two surrounding states, the risk of ILI in this area was 11.66 times more likely to develop the disease than other areas (LLR = 69,009, *p* < 0.001). Level 2, with Virginia at the center of a high incidence area and three surrounding states, the risk of ILI in this area was 9.79 times more likely to develop the disease than other areas (LLR = 73,277, *p* < 0.001). Level 3, with New Mexico at the center of high incidence area and three surrounding states, the risk of ILI in this area was 3.38 times more likely to develop the disease than other areas (LLR = 26,518, *p* < 0.001). At the same time, the states with a high cluster in the local spatial autocorrelation analysis were all located in the high cluster area, the results were consistent. From the cluster time, the high incidence time mainly occurs between January and March (Table 2).

### 3.3. Time-Series Analysis

Based on the result of spatiotemporal analysis, the HH cluster was mainly in Mississippi and Louisiana. In particular, Mississippi has been exhibiting an HH cluster in recent years. So we predict the incidence of ILI in Mississippi by time-series analysis.

**Figure 5:**
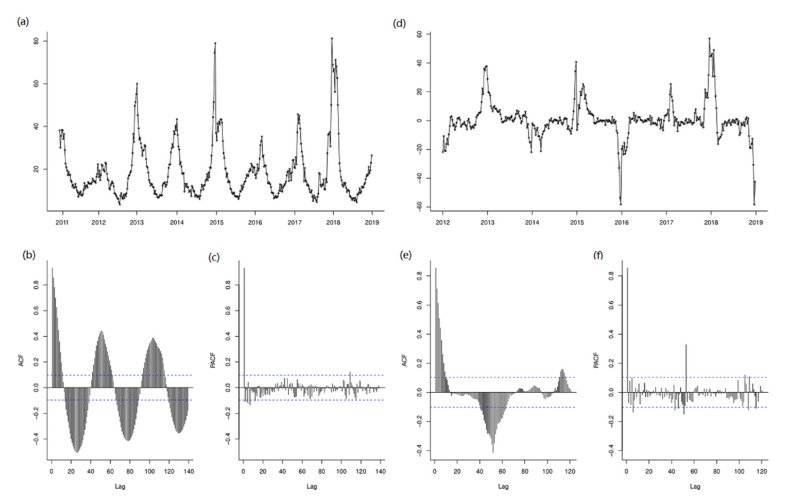
The time diagram, ACF and PACF graphs for estimating the parameter: (**a**) the time diagram of ILI incidence after one-order seasonal difference data, (**b**) the ACF graph of the raw data (d = 0, D = 0), (**c**) the PACF graph of the raw data (d = 0, D = 0), (**d**) the time diagram of ILI incidence a, (**e**) the ACF graph of one-order seasonal difference data (d = 0 and D = 1), (**f**) the PACF graph of one-order seasonal difference data (d = 0 and D = 1).

Using raw training data from 1st week 2011 to 52nd week 2018, the trend difference (d = 0) and seasonal difference (D = 1) were calculated (Figure 5). The augmented Dickey−Fuller Test indicated the sequence was stationary (*t* = −3.98, *p* = 0.01). The ACF and PACF plots were used to estimate the parameter ranges of p, P and q, Q [26]. After checking ACF and PACF plots, SARIMA (1, 0, 0) (1, 1, 0)_52_ was the best fitted model with lowest AIC and BIC values, and the Ljung−Box Q Test of this model is valid ($\chi^{2}$ = 21.822, *p* = 0.149), indicating it was a white noise sequence. All the parameter estimates were significant (Table 3).

**Figure 6:**
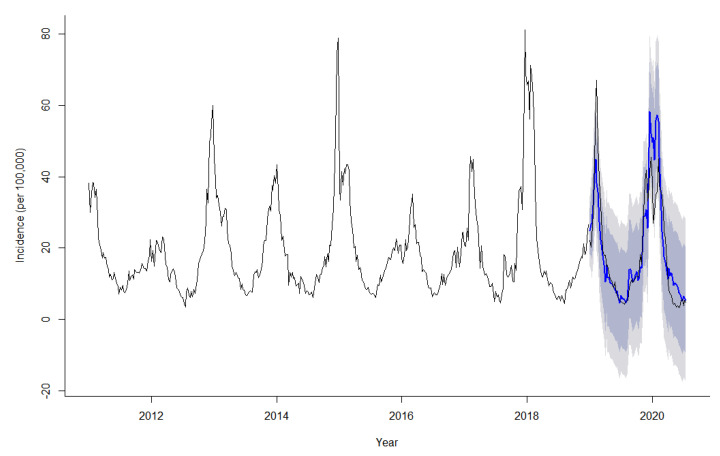
Comparison of actual and predicted incidence of ILI in the United States.

The model SARIMA (1, 0, 0) (1, 1, 0)_52_ forecasting effect was tested by comparing the predicted values with the observed values from 1st week 2019 to 29th week 2020. As Figure 6 shows, the black and blue lines represent the observed values and predicted values, respectively, and the dark gray and light gray represent 80% and 95% confidence intervals, respectively. The predicted trend of ILI incidence was basically consistent with the actual trend, and both the root mean squared error (RMSE) and mean absolute percent error (MAPE) were small, indicating that the model prediction results were reliable. Then, forecasting the ILI incidence from 30th week 2020 to 52nd week 2021 by SARIMA. The forecast results showed that there was a high ILI incidence in winter and spring, and low ILI incidence in summer and autumn. The incidence of ILI will reach its peak in the 6^th^ week 2021 (Table 4).

## 4. Discussion

Influenza is a contagious respiratory illness caused by influenza viruses. It can cause mild to severe illness. Serious outcomes of influenza infection can result in hospitalization or death. Due to the low detection rates, it is easy to underestimate the severity of influenza. The weekly ILI tests conducted by the CDC can effectively remind us of influenza trends. The main purpose of this study was to explore the epidemiological characteristics of ILI incidence, identify the states and possible clusters with high ILI incidence in the United States through spatiotemporal analysis, and then construct a SARIMA model to realize the short-term prediction of ILI incidence.

In the descriptive analysis, we recorded age characteristics, seasonal peaks and regional differences. Those individuals most at risk for severe symptoms and complications from this virus are the very young, vulnerable older adults, pregnant women, immunocompromised individuals of all ages, and those with chronic comorbid conditions [27]. Many studies indicated that influenza viruses caused severe morbidity and mortality in the elderly [28]. However, in this study, people aged 65 and over accounted for the lowest proportion of ILI cases. One of the reasons is that the cardinal number of population of 65 years and over is small. During the study period, a gradual increase in ILI incidence was observed, particularly during the 2018 and 2019 influenza seasons, with a sharp increase in the incidence of ILI. There are many reasons to explain the influenza outbreaks. Both extreme weather and insufficient vaccines are important reasons that affect the incidence of influenza [29].

Spatiotemporal analysis was used to identify high-risk areas for multiple diseases [20]. Yue [30] and Freitas [31] used spatiotemporal analysis to identify the spatial clustering characteristics of dengue fever cases. Liu [23] used spatiotemporal scanning analysis to explore the high-risk areas of hand, foot and mouth disease. In this study, the high incidence of influenza was mainly concentrated in the states of Louisiana, Virginia and Mississippi. Spatiotemporal analysis revealed the HH clusters and high-risk states were mainly located in Mississippi, and the time clusters were mainly concentrated in January to March. This finding was confirmed by other studies [32,33]. Time-series analysis has the advantage of predicting the incidence. It is characterized by the number of patients in the past and responds by predicting the number of patients in the future [13]. The prediction showed that the 95% confidence interval of the predicted ILI incidence almost contained the observed value. The RMSE and MAPE were small, which supported that the SARIMA model was effective in the prediction of ILI. Then, we used this model to forecast the ILI incidence from 30^th^ week 2020 to 52^nd^ week 2021. The results demonstrated that ILI incidence will increase in 45^th^ week 2020 and peak in 6^th^ week 2021, and the distribution is similar to the previous years.

Influenza viruses spread through human contact. Therefore, geography and population density are potential factors of influenza transmission [34]. According to Garrett’s research, the high population density will accelerate the spread of influenza [35]. In this study, the Northeast and Southwest were the most densely populated areas with the lower ILI incidence in the United States. Mississippi is a mostly rural state with a low population density and the highest incidence, different from the results of Garrett’s research [35]. The reason for the results of this study might be the lower economic level of Mississippi [35,36]. During epidemics, the poorest part of the population usually suffers the most. In addition, Mississippi is also the state with the highest proportion of black Americans. Many studies have shown that black individuals have a higher proportion of influenza cases [37,38,39].

Transmission of influenza varies across seasons and geographical areas in the United States. The obvious temporal clusters during the winter and spring, which was in accordance with the seasonality of the respiratory disease [40]. Most parts of the United States are temperate or subtropical climate. The continental climate zone in the central plain is characterized by the cold winters. Winters in temperate regions are characterized by an average temperature between 0 °C and 20 °C, with the minimum temperature dropping to as low as −40 °C in some regions. Generally speaking, influenza peaks in temperate regions in winter. However, the seasonal pattern in subtropical regions seem to be more complicated [29]. Influenza transmission is influenced by variations meteorological variables such as temperature, absolute humidity and precipitation. In the annual influenza epidemics of the United States, the transmission of influenza increases during periods with low precipitation and absolute humidity [41]. Absolute humidity is the total water content in the air. The survival rate of the influenza virus increases at lower absolute humidity levels. Relative humidity increases with the high precipitation. High relative humidity will accelerate the accumulation of respiratory droplets, which reduces the spread of influenza virus. In contrast, low relative humidity is favorable to the spread of influenza virus. The suitable temperature range activating influenza viral transmission could partially explain the common winter epidemics in the central regions [42].

## 5. Limitations

There is need to highlight some limitations that may be associated with our study outcomes. First, since all influenza activity reporting by public health partners and health-care providers was voluntary, it was difficult to maintain the quality and consistency of the source of data. Second, the influenza incidence of 2020 may be underestimated due to the masks. Therefore, the 2020 data was used for model testing instead of model building. Third, the influence factors of influenza activity were not studied in-depth in this study. In the next step, the risk factors need being explored.

## 6. Conclusions

In this study, we found the high-risk clusters were concentrated in the southeast, and the incidence of influenza may reach its peak in the 6th week 2021. In order to limit the spread of the outbreaks, surveillance activities and health education should be selectively carried out in higher incidence areas in the epidemic season.

## Figures and Tables

**Table 1:** Global spatial autocorrelation analysis.

Year	Moran’s I	E(I)	Mean	S	Z-Value	*p*-Value
2011	0.099	−0.021	−0.023	0.090	1.358	0.093
2012	0.185	−0.021	−0.019	0.095	2.151	0.028
2013	0.181	−0.021	−0.019	0.094	2.121	0.031
2014	0.200	−0.021	−0.021	0.092	2.401	0.022
2015	0.166	−0.021	−0.222	0.091	2.073	0.037
2016	0.177	−0.021	−0.024	0.093	2.150	0.029
2017	0.146	−0.021	−0.025	0.087	1.981	0.039
2018	0.074	−0.021	−0.023	0.092	1.053	0.145
2019	0.074	−0.021	−0.021	0.090	1.053	0.139

Year: “year” represents 1st to 52nd week of each year.

**Table 2:** Spatiotemporal scan of ILI in the United States from 2011 to 2019.

Year	Level	Center	N	Cluster Period	Coordinates/Radius(km)	ObservedCases	ExpectedCases	RR	LLR	*p*-Value
2011	1	Kentucky	15	2011-01-01 to 2011-02-28	(37.5 N, 85.3 W)/738.2	121,829	24,485	6.22	108,601	<0.001
	2	Colorado	13	2011-01-01 to 2011-02-28	(39.0 N, 105.5 W)/1005.2	62,527	15,984	3.91	4.32	<0.001
2012	1	Mississippi	3	2012-10-01 to 2012-12-31	(32.8 N, 89.7 W)/289.2	37,831	4698	8.65	46,953	<0.001
	2	Virginia	1	2012-09-01 to 2012-12-31	(37.5 N, 78.8 W)/0.0	29,130	4234	7.26	31,944	<0.001
	3	Nebraska	17	2012-01-01 to 2012-03-31	(41.5 N, 99.8 W)/1116.02	65,301	32,693	2.15	13,777	<0.001
2013	1	Virginia	3	2013-01-01 to 2013-03-31	(37.5 N, 78.8 W)/222.1	46,638	4750	10.61	66,247	<0.001
	2	Texas	15	2013-01-01 to 2013-2-28	(31.5 N, 99.4 W)/1327.5	101,773	27,333	4.32	64,734	<0.001
2014	1	Mississippi	3	2014-10-01 to 2014-12-31	(32.8 N, 89.7 W)/289.2	45,125	5686	8.52	55,411	<0.001
	2	Virginia	3	2014-01-01 to 2014-04-30	(37.5 N, 78.8 W)/222.1	43,127	6524	7.06	46,035	<0.001
	3	New Mexico	12	2014-01-01 to 2014-02-28	(34.4 N, 106.1 W)/1249.5	54,639	18,361	3.18	24,494	<0.001
2015	1	Louisiana	2	2015-01-01 to 2015-04-30	(31.1 N, 92.0 W)/289.2	45,837	4254	11.66	69,009	<0.001
	2	Virginia	3	2015-01-01 to 2015-04-30	(37.5 N, 78.8 W)/222.1	54,666	6134	9.79	73,277	<0.001
	3	New Mexico	12	2015-01-01 to 2015-02-28	(34.4 N, 106.1 W)/1249.5	54,311	17,264	3.38	26,518	<0.001
2016	1	Virginia	3	2016-01-01 to 2016-05-31	(37.5 N, 78.8 W)/222.1	63,287	7974	8.81	78,624	<0.001
	2	Arizona	3	2016-01-01 to 2016-04-30	(34.3 N, 111.7 W)/559.1	32,319	7155	4.73	24,144	<0.001
	3	Mississippi	10	2016-01-01 to 2016-04-30	(32.8 N, 89.7 W)/820.6	104,314	34,608	3.47	50,171	<0.001
2017	1	Florida	13	2017-01-01 to 2016-03-31	(28.6 N, 82.5 W)/1247.2	192,625	51,339	4.63	127,775	<0.001
	2	Oregon	1	2017-11-01 to 2017-12-31	(43.9 N, 120.6 W)/0.0	6819	1714	4.01	4329	<0.001
	3	Colorado	14	2017-01-01 to 2017-02-28	(39.0 N, 105.5 W)/1024.3	54,139	25,185	2.23	13,031	<0.001
2018	1	Florida	13	2018-01-01 to 2018-02-28	(28.6 N, 82.5 W)/1247.2	259,855	47,137	6.89	253,645	<0.001
	2	Wyoming	13	2018-01-01 to 2018-02-28	(43.0 N, 107.6 W)/1052.7	74,812	19,512	4.04	46,665	<0.001
2019	1	Colorado	7	2019-01-01 to 2019-03-31	(39.0 N, 105.5 W)/747.6	82,746	19,164	4.52	58,874	<0.001
	2	Florida	13	2019-01-01 to 2019-03-31	(28.6 N, 85.5 W)/1247.2	326,625	94,525	4.16	193,772	<0.001

RR: risk ratio; LLR: logarithmic likelihood ratio.

**Table 3:** Comparison of candidate SARIMA models.

Model	Estimate	t	*p*	Ljung-Box Q Test		AIC	BIC	RMSE	MAPE
				Statistics	*p*				
SARIMA (1, 0, 0) (1, 1, 0)_52_	-	-	-	21.822	0.149	2235.530	2247.220	4.673	14.290
AR1	0.886	36.768	<0.001	-	-	-	-	-	-
SAR1	−0.607	14.350	<0.001	-	-	-	-	-	-
SARIMA (1, 0, 1) (1, 1, 0)_52_	-	-	-	20.962	0.138	2235.110	2250.700	4.655	14.368
AR1	0.865	28.837	<0.001	-	-	-	-	-	-
MA1	0.097	1.5410	0.065	-	-	-	-	-	-
SAR1	−0.612	−14.495	<0.001	-	-	-	-	-	-
SARIMA (2, 0, 0) (1, 1, 0)_52_	-	-	-	20.734	0.146	2201.100	2216.700	14.390	0.970
AR1	0.957	18.254	<0.001	-	-	-	-	-	-
AR2	−0.080	−1.511	0.067	-	-	-	-	-	-
SAR1	−0.612	−14.495	<0.001	-	-	-	-	-	-
SARIMA (2, 0, 1) (1, 1, 0)_52_	-	-	-	18.552	0.183	2233.530	2253.012	4.636	14.602
AR1	0.131	0.695	0.245	-	-	-	-	-	-
AR2	0.653	3.744	<0.001	-	-	-	-	-	-
MA1	0.835	5.088	<0.001	-	-	-	-	-	-
SAR1	−0.605	14.157	<0.001	-	-	-	-	-	-
SARIMA (2, 0, 2) (1, 1, 0)_52_	-	-	-	18.405	0.143	2233.480	2256.87	4.599	14.906
AR1	−0.080	−2.161	0.018	-	-	-	-	-	-
AR2	0.825	26.101	<0.001	-	-	-	-	-	-
MA1	1.064	16.272	<0.001	-	-	-	-	-	-
MA2	0.064	0.994	0.163	-	-	-	-	-	-
SAR1	−0.611	−14.45	<0.001	-	-	-	-	-	-

AIC: Akaike information criterion; BIC: Bayesian information; RMSE: root mean squared error; MAPE: mean absolute percent error.

**Table 4:** Predictive value of ILI incidence (per 100,000).

Year/Week	Incidence	95% CI	Year/Week	Incidence	95% CI
2020/30	5.504	−4.248–15.256	2021/16	11.536	−8.684–31.755
2020/31	5.163	−7.801–18.128	2021/17	10.344	−9.876–30.563
2020/32	4.681	−10.288–19.651	2021/18	8.225	−11.995–28.444
2020/33	6.943	−9.399–23.284	2021/19	7.892	−12.327–28.112
2020/34	8.422	−8.900–25.743	2021/20	7.633	−12.587–27.853
2020/35	9.549	−8.488–27.587	2021/21	6.467	−13.753–26.687
2020/36	11.084	−7.484–29.652	2021/22	7.606	−12.613–27.826
2020/37	9.433	−9.532–28.398	2021/23	5.670	−14.550–25.890
2020/38	9.993	−9.271–29.258	2021/24	5.411	−14.809–25.63
2020/39	11.696	−7.795–31.187	2021/25	5.784	−14.436–26.004
2020/40	11.710	−7.953–31.373	2021/26	5.468	−14.751–25.688
2020/41	11.726	−8.068–31.520	2021/27	4.498	−15.721–24.718
2020/42	12.617	−7.276–32.511	2021/28	4.990	−15.230–25.21
2020/43	15.611	−4.359–35.581	2021/29	4.672	−15.548–24.892
2020/44	15.406	−4.623–35.434	2021/30	4.823	−15.851–25.496
2020/45	20.754	0.681–40.827	2021/31	5.010	−16.006–26.025
2020/46	23.021	2.913–43.128	2021/32	4.910	−16.364–26.184
2020/47	26.565	6.431–46.698	2021/33	6.056	−15.414–27.526
2020/48	30.270	10.116–50.423	2021/34	8.693	−12.926–30.313
2020/49	24.789	4.619–44.958	2021/35	9.995	−11.739–31.728
2020/50	26.030	5.849–46.211	2021/36	11.233	−10.587–33.054
2020/51	31.144	10.954–51.334	2021/37	9.925	−11.962–31.812
2020/52	34.819	14.622–55.016	2021/38	10.378	−11.560–32.316
2021/01	29.301	9.099–49.503	2021/39	11.596	−10.380–33.573
2021/02	23.157	2.951–43.363	2021/40	11.996	−10.010–34.003
2021/03	25.995	5.785–46.204	2021/41	11.853	−10.176–33.883
2021/04	32.197	11.985–52.409	2021/42	12.471	−9.577–34.518
2021/05	40.479	20.266–60.693	2021/43	17.086	−4.975–39.147
2021/06	54.811	34.596–75.026	2021/44	15.498	−6.574–37.569
2021/07	54.064	33.848–74.28	2021/45	23.641	1.562–45.720
2021/08	43.281	23.064–63.498	2021/46	26.943	4.858–49.028
2021/09	37.155	16.938–57.373	2021/47	33.262	11.172–55.352
2021/10	31.506	11.288–51.724	2021/48	36.767	14.674–58.86
2021/11	24.705	4.487–44.924	2021/49	29.557	7.461–51.654
2021/12	20.839	0.620–41.058	2021/50	30.704	8.606–52.802
2021/13	17.175	−3.044–37.394	2021/51	36.928	14.828–59.027
2021/14	14.670	−5.549–34.889	2021/52	40.739	18.638–62.84
2021/15	11.900	−8.319–32.119			

## Data Availability

The data presented in this study are openly available in the Centers for Disease Control and Prevention (CDC; https://www.cdc.gov/flu/weekly/, accessed on 24 November 2020).
